# Editorial

**Published:** 1866-03

**Authors:** 


### Editorial.

MEDICAL COLLEGE INSTRUCTION.—The regular clinical and reading term in the Chicago Medical College, will commence on the first Tuesday in March, and continue until the first of July. The course of instruction will consist of one clinic on Mondays, Tuesdays, Thursdays, and Fridays, in Mercy Hospital, and on Wednesdays and Saturdays in the College Dispensary, making one clinical lecture every day as a part of the regular course. In addition, the student will have free access to the clinical instruction given in the County Hospital, which is within five minutes walk from the College. Besides this ample provision for clinical instruction, at least one familiar lecture and examination will be given each day, by the members of the College Faculty. Ample material will also be furnished to those who wish to pursue dissections.

The Spring and Summer Term, as thus arranged, affords very superior advantages to all such students as can afford to remain in the city until July. It ensures a systematic course of practical and demonstrative instruction of the most valuable character, without the crowding of many topics upon the mind the same day, as in the regular winter term. It is desirable that it should be distinctly understood, however, that this summer reading and clinical term is merely supplementary to the annual lecture term, and in no case to be counted as *one college term*

by the student in making up his requirements for graduation. It has been no part of the objects of the Faculty of the Chicago Medical College to increase the *facilities* for the graduation of students, by making two lecture terms in one year, or by adhering to one short and wholly inadequate term. On the contrary, their great leading object is to induce medical students to take a higher and broader course of medical instruction, before attempting to graduate. The Summer Course is free to all the matriculants of the College.

---

County Hospital.—At a regular meeting of the Medical Board of the County Hospital, located in this city, it was decided to open the institution, at once, for clinical instruction. For eight months of the year—from Oct. 1st to June 1st—there will be given four clinics a week, two medical and two surgical. For the remaining four months, viz.:—from June 1st to Oct. 1st, there will be two clinics a week, one medical and one surgical. The clinic days are Tuesdays and Fridays, at 1½ o'clock P.M., which will be observed throughout the entire year.

The profession of the city and country are invited to visit the hospital at any time, and especially on clinic days.

The following gentlemen have been elected and duly installed in the duties of their office:—E. T. Quales, M.D., House Physician, W. H. Hutchinson, Assistant.

---

Chicago Medical College Annual Commencement.—The Annual Commencement exercises of the Chicago Medical College were held March 1st, 1866, in the Hall of the College, No. 1015 State Street. A large number of the relatives and friends of the graduating class were present, filling the Hall to its utmost capacity.

The exercises of the evening were opened by prayer by Rev. Mr. Trowbridge.

*The Graduating Class.*—The President, Dr. H. A. Johnson then conferred the degree of Doctor of Medicine upon the members of the graduating class, which was composed of the following named gentlemen:—

J. N. Bishop, H. W. Boyd, James Brewster, W. H. Buchtel, D. F. Crouse, J. W. Filkins, C. Hamilton, H. Harris, W. Horne, D. S. Jenks, C. T. Johnson, J. F. Kelsey, E. A. Lee, J. McCarthy, H. C. McCoy, S. A. McWilliams, W. E. Morris, W. A. Nason, Henry Shimer, Lyman Ware, N. W. Webber, Herbert York.

*Ad Eundem*—Drs. W. H. Baxter, W. S. Caldwell, George H. Calkins, W. D. Carter.

Honorary—Drs. J. Charlton, S. France, W. C. Matchette, J. P. Randall, L. D. Robinson.

The prizes for the best theses were conferred on S. A. McWilliams and Henry Shimer.

*Address of the President.*—After the class had received their diplomas, the President addressed them as follows:—

In the early history of the medical profession it was customary to require candidates for medical honors to take an obligation to faithfully perform the duties of their profession. We have not required of you, gentlemen, formally, this obligation; but in conferring upon you the degree of doctor of medicine the College is in the most emphatic manner indorsing your professional character, and recommending you to the public confidence. In accepting this degree, you are under the implied obligation no less binding than the Hyppocratic oath, to faithfully and truly perform the duties of the high calling to which you have this evening, consecrated your lives. You are virtually pledged to a life-long service in the fields of science and art. You have promised to conscientiously use the power and influence of your profession for relieving suffering, and for the promotion of the best interests of humanity. In the name of the College, then, I charge you to cultivate science with an earnest desire to know the truth and to practice the art of medicine and surgery, ever remembering its legitimate use—the relief of suffering and the lengthening, if possible, of human life. And, finally, to cherish in your own hearts every social virtue, loving purity, goodness, and truth; so shall your lives be honored and your memories cherished.

*Award of Prizes.*—Dr. N. S. Davis then presented to Messrs

S. A. McWilliams and Henry Shimer the prizes for having written the best theses in the class. Mr. S. A. McWilliams received the first prize, which consisted of a photographic likeness of the donor, Dr. Davis. Mr. Shimer received the second prize, a copy of the transactions of the Chicago Medical Society from the year 1856 to 1864.

*The Valedictory.*—Prof. Wm. H. Byford then delivered the valedictory address to the graduating class. The subject of the lecture was "The Philosophy of Trades and Professions." The lecturer said that the great end of learning trades and adopting professions was to get money. The great aims were selfish; but the professions of medicine and divinity were exceptions, for the practitioners in both these professions aimed at doing good. The physician could not do like the tradesmen,—"get all he can and keep all he gets," but his services were paid for according to the generosity of the people.

The speaker then proceeded to show the relation that the physician bore to the other two learned professions. The three learned professions had their foundation in the weaknesses of mankind. The legal profession was brought into existence becausethere were in the world weak men and rascals. The doctor had to deal with the bodily ills of mankind, and the clergy had the moral evils of the world to repair. The lecturer proceeded to show the vast field of knowledge open to the medical practitioner, and advised each one of the class to turn his attention particularly to some branch of the profession, since it was impossible that one person should be profound in them all.

He closed by thanking the class for their uniform diligence and good conduct while members of the Institution, and hoped they would all honor the profession they had adopted, and the College from which they received their diplomas.

At the close of the exercises, the President announced that the graduating class, together with their friends, would repair to the residence of Dr. Hollister, No. 30 Washington Street, where a bountiful supper would be served.

The meeting then adjourned.

At the residence of Prof. Hollister, the company enjoyed not only an elegant entertainment, but a most delightful social conference, which was not terminated until a late hour of the night.

---

Chicago Medical Society.—*Pathological Specimens and Cases.*—At the regular meeting of this Society, held February 9th, 1866, Dr. N. S. Davis presented the heart of a patient, whose history was briefly as follows:—About eight months previous to his death, he was thoroughly exposed to cold and wet, resulting in an attack of rheumatic fever that continued several weeks. After the fever subsided, he so far regained his health as to be able to do some work, but never fully recovered. His breathing was embarrassed whenever he took exercise, and about six weeks before his admission into Mercy Hospital his feet and ankles began to be œdematous, and his respiration more oppressed. The œdema increased rapidly, and at the time of his admission, which was the first of his coming under the observation of Dr. Davis, the superficial areolar tissue, not only of the upper and lower extremities, but through the whole extent of the body, was thoroughly œdematous, giving to the patient a very bloated aspect. His respiration was extremely difficult, accompanied by a mixture of dry, wheezing, and sharp submucous rhonchi through the whole extent of the chest, with decided dulness over the lower and posterior parts of the lungs. His countenance was pale, bloated, and expressive of anxiety; pulse feeble, frequent, and intermitting; the impulse of the heart feeble, and the systole accompanied by a loud, harsh, and prolonged bellows murmur, most intense one inch directly below the nipple, and a slighter rough sound with the diastole; urine very scanty and highly albuminous; little or no appetite, and bowels inclined to constipation. The case was made the subject of a clinic, and each member of the class in attendance had an opportunity to listen to the sounds of the heart and lungs.

The diagnosis was, extensive thickening of the valves in the left cavities of the heart, from previous endocardial inflammation; renal congestion with universal anasarca; and some œdema of the lungs, the latter threatening to terminate the life of the patient by suffocation in a very short time. As was anticipated, the pulmonary œdema rapidly increased, and the patient died on the third day after admission. The heart, which was the only part presented to the Society, was moderately increased in size as a whole, and covered with more fatty tissue than natural. There were no external traces of inflammation. On opening the cavities of the right side, the appearances were strictly natural. Both the auriculo-ventricular and the polmonary artery valves were perfectly free from any abnormal changes. On opening the left ventricle its whole interior surface was red and roughened, from previous inflammation. The columnæ carnæ were unusually thick and their surfaces rough, and the shape of the cavity somewhat altered. It seemed to be contracted at the base, and enlarged, so as to appear slightly saculated, at the apex. At the base the walls were thick, but thinner than natural towards the apex. The mitral valve was extremely thickened and so changed in color and texture as to resemble the columnæ carnæ in appearance, and almost as hard as cartilage. The thickening of this valve had so narrowed the opening from the left auricle to the left ventricle that it would not admit the end of the little finger. The semi-lunar valves of the aorta were also so much thickened and indurated as to present quite a serious obstruction to the egress of blood from the ventricle. These changes fully explained the constant fulness of the pulmonary veins and the consequent capillary congestion, dyspnœa, with final pulmonary œdema and death.

Dr. T. D. Fitch presented the morbid specimens obtained from the base of the cranium and membranes of the brain, belonging to the case related to the Society at a previous meeting, and reported in the Examiner for February.

Dr. J. P. Ross related a very interesting case of extreme abdominal tympanites, ending in pneumothorax, with emphysema at the top of the chest, and death. The case was a laboring man, who had complained of some gastric symptoms, or indigestion, for one or two years, but not so severe as to prevent his regular labor, and the day previous to the attack had been at his work and as cheerful as ever. On his way home at evening, he was attacked with severe pain in the stomach, and stopped at a drug-store, obtained a dose of cathartic pills, and took them. They did not operate, however, and, before morning, the pain in the epigastric and left hypochondriac regions became so intense as to be scarcely endurable. Dr. CLARK was called very early in the morning, and prescribed full doses of anodynes internally and fomentations externally. At that time, there was no tympanites; no febrile reaction; and the pain was still restricted to the region of the stomach. About the middle of the afternoon, however, Dr. ROSS was called in, and found the patient still in very great distress, and with an extraordinary degree of tympanitic distension of the abdomen. The pressure against the diaphragm was sufficient to render respiration very short, and the abdominal walls were, literally, as tense as a well-corded drum. On attempting to give an enema, it was found impossible to force anything into the rectum. In a few minutes after, while being turned partly upon one side, the patient felt something give way, with a sound audible to the by-standers, and in a moment the respiration became so greatly oppressed as to threaten immediate suffocation. The whole left side of the chest became almost as tympanitic as the abdomen, and an emphysematous swelling appeared in the space above the clavicle. In a few moments, the patient died, apparently from suffocation.

A strong effort was made to procure the privilege of making a *post mortem* examination, but without success. It would seem probable, from all the symptoms, that perforation of the stomach took place at the time the intense pain commenced; that the enormous accumulation of gas which was in the peritoneal sac, by its pressure, not only greatly distended the abdominal parieties and collapsed the intestines, but finally forced a passage through the diaphragm, filling the left side of the chest and following the areolar tissue of the mediastinum to the top of the chest, presenting there an emphysematous tumor above the clavicle. It was certainly an extraordinary case, and the refusal of friends to allow a proper *post mortem* examination deserves severe censure.

Death of Dr. I. P. Lynn.—At a meeting of the profession, held at the Council Chamber, Chicago, March 2d, 1866, on the occasion of the death of Dr. I. P. Lynn, Dr. R. C. Hamill was elected Chairman, and Dr. Thos. Bevan, Secretary.

On motion, Drs. Allen, Blake, and Miller were appointed a committee to draft suitable resolutions in relation to the melancholy event.

Dr. E. Ingalls made a brief address, eulogistic of the estimable qualities of our departed confrére.

Dr. J. A. Allen, from the Committee on Resolutions, reported as follows:—

*Resolved*, That the intelligence of the sudden and untimely death of our late friend and co-worker in the medical profession, Isaiah P. Lynn, M.D., is received by this meeting with emotions of profound pain and sincere sorrow.

*Resolved*, That the memory of our deceased brother will be cherished by us as of one who, personally, and by his amiable disposition and fine friendly feeling, endeared himself to all who knew him intimately; whilst, by his conscientious earnestness in acquainting himself with the principles of medical science, and thorough devotedness to the welfare of those who entrusted themselves to his care as patients, he commanded the confidence, the respect, and esteem of both the profession and the public.

*Resolved*, That we tender to his bereaved family and friends, the assurances of our warmest sympathy in this their great affliction.

*Resolved*, That copies of these resolutions be presented to the relatives, the public press, and medical journals of this city for publication.

On motion, the report was accepted and adopted unanimously.

On motion of Dr. Hay, it was resolved that the profession attend the funeral in a body.

Dr. J. A. Allen then occupied the meeting with appropriate remarks, highly creditable to the amiable character, kindness, and faithfulness of the lamented dead. He also referred to the peculiarly sad circumstances of the immediate cause of death, giving an entirely satisfactory explanation of the accident which had resulted so unfavorably.

Dr. V. L. Hurlbut followed Dr. Allen in a similar vein.

Drs. Brooks and McVickar referred feelingly to the courtesy and worth of the deceased, Dr. M. giving some incidents of his personal relations to the Doctor, of a very pleasant character.

Drs. N. S. Davis, S. Wickersham, and D. L. Miller also addressed the meeting, expressing their cordial endorsement of the resolutions, and of the sentiments of sympathy and respect which had been expressed by other speakers.

On motion, the meeting adjourned.

THOS. BEVAN, M.D., *Sec'y.*

---

Chicago Medical Journal.—This medical periodical has changed hands, editorially, and at the same time improved its typographical appearance. We wish its new editors much enjoyment in their editorial labors and abundant success.

---

### AMERICAN MEDICAL ASSOCIATION.

The Seventeenth Annual Session will be held in the City of Baltimore, on Tuesday, May 1, 1866.

The following Committees are expected to report:—

On Prize Essays, Dr. Austin Flint, Sr., N.Y., Chairman.

On Quarantine, Dr. Wilson Jewell, Pa., Chairman.

On So-called Spotted Fever, Dr. Jas. J. Levick, Pa., Ch'n.

On Ligature of the Subclavian Artery, Dr. Willard Parker, N.Y., Chairman.

On Tracheotomy in Membranous Croup, Dr. Alex. N. Dougherty, N.J., Chairman.

On Rank of Medical Corps in the Army, Dr. C. S. Tripler, U.S.A., Chairman.

On Rank of Medical Corps in the Navy, Dr. T. L. Smith, N.Y., Chairman.

On Medical Literature, Dr. C. A. Lee, N.Y., Chairman.

On Medical Education, Dr. Samuel D. Gross, Pa., Chairman.

On American Necrology, Dr. C. C. Cox, Md., Chairman.

On Patent Rights and Medical Men, Dr. David Prince, Ill., Chairman.

On Alcohol and its Relations to Man, Dr. Gerard E. Morgan, Md., Chairman.

On Insanity, Dr. Alfred Hitchcock, Mass., Chairman.

On Milk Sickness, Dr. Robert Thompson, Ohio, Chairman.

On the Relation which the Doctrine of the Correlation and

Conservation of Forces bears to the Physiological and Pathological Condition of the Human System, Dr. S. L. Loomis, D.C., Chairman.

On the Progress of Medical Science, Dr. Jerome Candee Smith, N.Y., Chairman.

On Diphtheria, Dr. H. D. Holton, Vt., Chairman.

On the Comparative Value of Life in City and Country, Dr. Edw. Jarvis, Mass., Chairman.

On Drainage and Sewerage of Cities in their Influence on Health, Dr. Wilson Jewell, Pa., Chairman.

What Effect has Civilization on the Duration of Human Life, Dr. Augustus A. Gould, Mass., Chairman.

On Disinfectants, Dr. E. M. Hunt, N.J., Chairman

On Compulsory Vaccination, Dr. A. Nelson Bell, N.Y., Ch'n.

On Strangulated Hernia, Dr. W. F. Peck, Iowa, Chairman.

On the Causes and Pathology of Pyæmia, Dr. J. J. Woodward, U.S.A., Chairman.

On the Use of Plaster of Paris in Surgery, Dr. Jas. L. Little, N.Y., Chairman.

On the Etiological and Pathological Relations of Epidemic Erysipelas, Spotted Fever, Diphtheria, and Scarlatina, Dr. N. S. Davis, Ill., Chairman.

On Meteorology, Medical Topography, and Epidemics, Drs. J. C. Weston, Me.; P. A. Stackpole, N.H.; C. L. Allen, Vt.; A. C. Garratt, Mass.; C. W. Parsons, R.I.; B. H. Catlin, Ct.; E. M. Chapman, N.Y.; E. M. Hunt, N.J.; D. Francis Condie, Pa.; T. Antisell, D.C.; O. S. Mahon, Md.; T. M. Logan, Cal.; R. C. Hamill, Ill.; J. W. H. Baker, Iowa; Abm. Sager, Mich.; J. W. Russell, Ohio. WM. B. ATKINSON.

*Permanent Secretary, Philadelphia.*

---

Increased Use of Stimulants in the London Hospitals. —Some remarkable statistics, regarding the employment of stimulants and the mortality in the London Hospital during some past years, appear in the last volume of the *Reports* of that hospital. In 1862, the number of in-patients was 4519, and the general mortality 7.6 per cent. The quantity of stimulants consumed was 1281 gallons of wine, 162 gallons of brandy, 38 gallons of gin, and 1100 ounces of cinchonine.

In 1864, the number of patients was 4619, and the general mortality 10.5 per cent; the stimulants consumed by these being 1558 gallons of wine, 359 gallons of brandy, and 77 gallons of gin. But as a set-off, if it may so be called, 760 more leeches were employed during this year than the average for the five preceding years, viz.: 3840. However, here we have a great increase in the amount of stimulants consumed, and also a great increase in the mortality of 1864, as compared with that of 1862. We state the facts, let it be understood, without in any way pretending to connect them as cause and effect.

Other statistics, Dr. Fraser gives us under this head:—"From 1854 to 1858, the annual average quantity of wine employed by each physician was 12,803 ounces;" each physician having an annual average of 391 patients under treatment. The annual average mortality was 11.87 per cent. But from 1860 to 1864, the annual average quantity of wine employed by each physician was nearly quadrupled, being 48,136 ounces; his annual average number of patients was 413; and the annual average mortality was 12.65 per cent.

From 1854 to 1858, each surgeon employed annually 38,016 ounces of wine; his annual number of patients was 1036; and the annual average mortality 4.48 per cent.

From 1860 to 1864 (five years,) each surgeon employed an annual average of 142,951 ounces of wine (nearly four times more than in the previous years;) the annual number of patients under him was 1065; and the annual average mortality 6.65 per cent.

Hence, we have, in the practice of both physicians and surgeons, a distinct increase of mortality coincident with great increase in consumption of stimulants.

Dr. Fraser also tells us (referring to a former paper of his) that, in 1851, there were 4051 in-patients in the London Hospital; that in 1857, there were 3935 in-patients; and that the mortality was greater in 1857 as 8 to 6.5 per cent, although £962 more were spent in 1857 than in 1851 for articles of luxury.

It is curious to note, that the only comment which Dr. Fraser makes on the above remarkable statistics is this:—

"It is evident, that a steady rise in the employment of stimulants------is still going on; and whatever be the cause, we may rest assured that the practice is imperative and needful; for it would be a monstrous assumption that a whole staff could be blindly following an objectless routine."

Not a single word of comment does Dr. Fraser bestow on the constant fact of the coincident increase of the mortality!

The summary of these statistics stands thus:—

From 1854 to 1858, each physician employed 12,800 ounces of wine annually; the deaths being 11.88 per cent. From 1860 to 1865, he employed 48,136 ounces; the deaths being 12.65 per cent.

During 1854 to 1858, each surgeon employed annually 38,016 ounces of wine; the deaths being 4.48 per cent. During 1860 to 1864, he employed annually 142,951 ounces; the deaths being 6.65 per cent.

In 1862, the general mortality of the hospital was 7.4 per cent; the consumption of stimulants being 1281 gallons of wine, 162 of brandy, and 38 of gin.

In 1864, the mortality was 10.5 per cent; the quantity of stimulants consumed being 6558 gallons of wine, 359 of brandy, and 12 of gin.

We again repeat, well knowing the sad fallacies which are so often edited through an erroneous interpretation of statistics, that we do not pretend to connect the increase of deaths with the increase of stimulants consumed. But, when we reflect upon our modern advancement in medicine and surgery (especially as miscalled conservative,) when we think of our great modern hygienic efforts, we may fairly ask for some explanation of the fact of a general advance in the mortality of a London Hospital.—*Brit. Med. Jour.*, Dec. 9, 1865, *and Medical News.*

---

Vaccine Matter from the Cow.—M. Lanoix has read a paper on this subject before the Academy of Medicine at Paris. This physician, after studying the subject at Naples, is founding in the capital of France an establishment for such vaccination. In the paper it is stated that out of 820 revaccinations practised in different schools upon children from 7 to 13 years old 21 per cent. succeeded. The figures respecting a more advanced age are as follows:—From 14 to 20 years, 71 revaccinations, 31 effectual; from 30 to 40 years, 200 revaccinations, 97 effectual; from 40 to 55 years, 30 revaccinations, 7 effectual; from 50 to 60 years, 5 revaccinations, 2 effectual. The author considers that the transmission of vaccine matter from heifer to heifer is always possible, the quantity obtained being quite adequate to very numerous operations; that the matter does not lose in activity in passing through animals as it does in passing through human organisms; that vaccinations are always or almost always successful; the revaccination with animal matter succeed more frequently than with matter obtained from human beings; that vaccination with heifer matter is extremely easy; and that such vaccinations are highly useful in epidemics of small-pox, as larger supplies of vaccine matter may rapidly sent to extensive tracts of country.—*London Lancet.*

